# Gene Expression Profiles Deciphering Leaf Senescence Variation between Early- and Late-Senescence Cotton Lines

**DOI:** 10.1371/journal.pone.0069847

**Published:** 2013-07-29

**Authors:** Xiangqiang Kong, Zhen Luo, Hezhong Dong, A. Egrinya Eneji, Weijiang Li, Hequan Lu

**Affiliations:** 1 Cotton Research Center, Shandong Key Lab for Cotton Culture and Physiology, Shandong Academy of Agricultural Sciences, Jinan, Shandong, China; 2 Department of Soil Science, Faculty of Agriculture, University of Calabar, Calabar, Nigeria; New Mexico State University, United States of America

## Abstract

Leaf senescence varies greatly among genotypes of cotton (*Gossypium hirsutium* L), possibly due to the different expression of senescence-related genes. To determine genes involved in leaf senescence, we performed genome-wide transcriptional profiling of the main-stem leaves of an early- (K1) and a late-senescence (K2) cotton line at 110 day after planting (DAP) using the Solexa technology. The profiling analysis indicated that 1132 genes were up-regulated and 455 genes down-regulated in K1 compared with K2 at 110 DAP. The Solexa data were highly consistent with, and thus were validated by those from real-time quantitative PCR (RT-PCR). Most of the genes related to photosynthesis, anabolism of carbohydrates and other biomolecules were down-regulated, but those for catabolism of proteins, nucleic acids, lipids and nutrient recycling were mostly up-regulated in K1 compared with K2. Fifty-one differently expressed hormone-related genes were identified, of which 5 ethylene, 3 brassinosteroid (BR), 5 JA, 18 auxin, 8 GA and 1 ABA related genes were up-regulated in K1 compared with K2, indicating that these hormone-related genes might play crucial roles in early senescence of K1 leaves. Many differently expressed transcription factor (TF) genes were identified and 11 *NAC* and 8 *WRKY* TF genes were up-regulated in K1 compared with K2, suggesting that TF genes, especially *NAC* and *WRKY* genes were involved in early senescence of K1 leaves. Genotypic variation in leaf senescence was attributed to differently expressed genes, particularly hormone-related and TF genes.

## Introduction

Senescence is the age-dependent end of the life span. In plants, it is characterized by the visible yellowing of leaves that accompanies the mobilization of leaf nutrients to the reproductive structures. The yellowing of senescing leaves is correlated with a series of biochemical changes such as loss of chlorophyll contents, degradation of proteins and RNA, and a decline in photosynthetic activity. Because accelerated leaf senescence curtails carbon assimilation, plant growth and yield are reduced [1], [2]. As the final stage of plant development, senescence has a crucial impact on agriculture, especially in crop production where crop yield is enhanced by longer growth periods. As for cotton, senescence may occur too early or too late in the season due to environmental stresses or internal factors [3], [4]. Too early senescence of whole cotton plant is referred to as premature senescence, which has been occurring on an increasing scale since modern transgenic *Bacillus thuringiensis* Berliner (Bt) cotton (*Gossypium hirsutum* L.) cultivars were introduced for commercial production [5], [6]. Wright [7], [8] indicated that premature senescence frequently developed during the period of rapid boll filling and this reduced lint yield and fiber quality, thus constituting an important constraint to cotton yield and quality.

During leaf senescence, viability of cells within the leaf is actively maintained until maximum remobilization has occurred [9]. This requires meticulous control of each step of the process, regulated by internal and external signals via a series of interlinking signaling pathways involving gene expression changes and influenced by the balance of hormones and metabolites [10]. Thus, senescence is a very complex process involving the expression of thousands of genes and many signaling pathways [10]–[12]. Elucidation of the relative influences of each pathway and the crosstalk between them is crucial to identify the key regulatory genes that control senescence [10].

Plant hormones play key roles in responses to senescence. Senescence is accelerated by the hormones ethylene, abscisic acid (ABA), and jasmonic acid (JA) that mediate plant responses to biotic and abiotic stresses. Exogenous ethylene enhances visible leaf yellowing and several ethylene biosynthesis genes are up-regulated in senescing leaves [12]–[14]. Ethylene- insensitive mutants such as ethylene-resistant 1 (*etr1*) and ethylene-insensitive 2 (*ein2*) display delayed leaf senescence [15], [16]. Similarly, the exogenous application of ABA accelerates leaf senescence [14], [17] and the level of ABA also increases during senescence [18], [19]. In addition, exogenous methyl jasmonic acid has been reported to accelerate leaf senescence. The JA-insensitive mutant, coronatine insensitive 1 (*coi1*) fails to display JA-dependent senescence [20]. Elevated cytokinin levels accompany delayed senescence, and endogenous cytokinin levels decrease during leaf senescence [19], [21]. Ectopic overproduction of cytokinins was shown to delay leaf senescence in tobacco, petunia, cassava and lettuce [22]–[26]. The induction of the senescence programme is characterized by up-regulation of a set of signature genes that are referred to as Senescence Associated Genes (SAGs) which include genes encoding specific catabolic enzymes and transcription factors. Accordingly, leaf senescence induced by the hormones ABA, JA, and ethylene is characterized by the induction of the expression of some SAGs whereas the delay of senescence by cytokinin is characterized with reduced expression of some SAGs [14], [27].

Auxins play important roles in multiple aspects of plant development and growth, including apical dominance, vascular differentiation, and shoot elongation. However, the role of auxin in regulating senescence is not as clear as that of cytokinins, ABA, and ethylene. Several earlier studies revealed that exogenous application of auxin delayed leaf blade abscission in bean [28], [29]. The exogenous application of auxin represses the transcription of senescence-response gene *SAG12* [30], and mutation of the *ARF2* and *ARF1* can delay senescence and *SAG12* expression [31]–[33]. The dominant activation mutant, *yuc6-1D* and *35S:YUC6* transgenic plants, which have been shown to contain an elevated free IAA level and to display typical high-auxin phenotypes, exhibit a delayed senescence phenotype [34]. On the other hand, auxin can stimulate the biosynthesis of senescence-promoting hormones such as ethylene and ABA [35], [36]. Further, the concentration of free IAA in senescing leaves of Arabidopsis was 2-fold higher than in non-senescing leaves [37], suggesting that auxin might have a senescence-promoting effect or accumulate as a consequence of senescence.

Salicylic acid (SA) plays a key role as a mediator of plant stress responses, including disease and systemic acquired resistance. It was also reported that the SA-signaling pathway was active in the control of developmental senescence [38]. A transcriptome analysis in senescing Arabidopsis leaves from wild-type plants and SA-deficient *NahG* mutants revealed that many SAGs are dependent on the SA-signaling pathway [11] and many SA related genes were up-regulated during senescence [12]. The roles of GA in leaf senescence are still not clear. van der Graaff et al. [12] reported that GA 2-oxidase 2 (*AtGA2OX2*) that deactivates GA was up-regulated during leaf senescence, suggesting that at least some GAs are deactivated during *Arabidopsis* leaf senescence. Greenboim-Wainberg et al. [39] reported that SPY acts as both a repressor of GA responses and a positive regulator of cytokinin signaling and plays a central role in the regulation of GA/cytokinin cross talk during plant senescence. Brassinosteroids appear to promote developmental senescence, as mutants deficient in BR biosynthesis or the BR receptor *BRI1* have a retarded senescence progression [40]. Most of the BR related genes were up-regulated during senescence in *Arabidopsis* [12].

About 100 TFs belonging to the APETALA2, basic-leucine zipper, MYB, NAC (NAM/ATAF1, 2/CUC2), WRKY, zinc finger, and GRAS families are differentially regulated during leaf senescence [11]. Many of the TFs involved in the developmental processes also play diverse roles in hormone signaling, stress responses, and metabolism, suggesting that extensive signaling crosstalk exists between intrinsic aging programs and environmental stress signals [41]–[43]. The NAC TFs constitute one of the largest transcription factor families in plants and *Arabidopsis* genome contains about 106 NAC members involved in diverse developmental processes and biotic and abiotic stress responses [44], [45]. The NAC TFs also constitute a large fraction of senescence- regulated genes in many plant species [11], [46]–[48]. The *Arabidopsis NAP* (*NAC-LIKE, ACTIVATED BY AP3/PI*) gene is transcriptionally regulated during senescing of *Arabidopsis* leaves [49]. Overexpression of *NAP* can lead to premature senescence, whereas the *NAP*-deficient mutant exhibits delayed leaf senescence. Notably, *Os NAP* and *Pv NAP*, restore the delayed leaf senescence in the *At NAP*-deficient mutant, showing that the *NAP* genes are conserved in diverse plant species [49]. *At ORE1* and *At ORS1* have been identified as nonredundant positive regulators of senescence, because inhibiting them individually delays leaf senescence [48], [50]. ABA responsive NAC transcription factor *VNI2* (*VND-INTERACTING2*) can integrate ABA- mediated abiotic stress signals into leaf aging by regulating a subset of *COR* (*COLD-REGULATED*) and *RD* (*Responsive TO Dehydration*) genes [43]. In addition, spatial and temporal expression patterns of the *VNI2* gene are correlated with leaf aging and senescence. Accordingly, leaf aging was delayed in transgenic plants overexpressing the *VNI2* gene but significantly accelerated in a *VNI2*-deficient mutant [43]. A hydrogen peroxide induced NAC transcription factor *JUB1* gene was also reported to regulate Arabidopsis senescence [51]. Leaf aging was delayed in transgenic plants overexpressing *JUB1* gene but significantly accelerated in *JUB1* deficient mutant, suggesting that *JUB1* is a negative regulator of senescence [51].

Our previous study showed that removal of early fruiting branches delayed leaf senescence but main-stem girdling accelerated leaf senescence in cotton. Leaf cytokinin content was increased in fruit-removal plants but decreased in girdled plants; in contrast, the ABA content was decreased in fruit removal plants and increased in girdled plants [6], [52]. Our previous results also suggested that the cotton line, K1 senescence earlier than K2 due to its lower cytokinin and higher ABA contents [19], [52]. Although studies have indicated possible correlation of leaf senescence with cytokinins and ABA in cotton [6], [19], [52], the expression profiles of senescence-related genes, particularly hormone related and TF genes and their involvement in senescence regulation in cotton have not been documented. In the present study, we compared the difference between the transcriptomes of main-stem leaves obtained from an early senescence (K1) and a late senescence (K2) cotton using the Solexa sequencing system during leaf senescence. By investigating changes in the expression of genes that contribute to senescence, a number of candidate genes related to cotton senescence were identified.

## Materials and Methods

### Plant Material and Growth Conditions

An early-senescence (K1) and a late-senescence (K2) cotton line with the same genetic background, developed by the Cotton Research Center, Shandong Academy of Agricultural Sciences, Jinan, were used in the experiment. Our previous trial showed that both K1 and K2 grew and developed in a similar time-course and performed similarly in shoot and root weights, shoot: root ratio, and plant biomass. However, leaf senescence based on chlorophyll content and photosynthesis in the late season differed significantly between the two cotton lines [19]. Leaves of K1 do senesce earlier than those of K2 after flowering. So the fourth leaves from the apex of both lines at 110 DAP were selected for gene expression profiling.

Acid-delinted seeds of each transgenic cotton line were sown in plastic pots (50 cm in height and 40 cm in diameter) filled with fertile soil (1.2% organic matter, 500 mg kg^−1^ total N, 15 mg kg^−1^ available P, and 120 mg kg^−1^ available K) on April 20, 2012, and allowed to germinate and grow in a greenhouse (30/20°C). When the first fully expanded true leaf occurred, seedlings were thinned to one per pot and allowed to grow under natural environment conditions. Potted plants were watered to 75% of the field water capacity daily to minimize water stress, and fertilized with 5 g compound fertilizer (25% N, 25% P, and 20% K) per pot at flowering stage. The fourth leaves from the apex on the main-stem of both lines were harvested separately at 65, 80, 95 and 110 DAP, frozen in liquid nitrogen and stored at −80°C for RNA extraction.

### RNA Extraction

Total RNA was extracted using the TRIzol reagent (Invitrogen), and mRNA was isolated from total RNA using Dynabeads Oligo (dT) (Invitrogen Dynal), following the manufacturer’s instructions. Dried RNA samples were dissolved in diethylpyrocarbonate- treated H_2_O, and the concentration determined spectroscopycally. The quality of the RNA was checked on 1.0% denaturing agarose gels. For Solexa sequencing, total RNA from 6 representative individual plants of each line was mixed together.

### Solexa Sequencing

At least 20 µg of total RNA was sent to Beijing Genomics Institute for Solexa sequencing (commercial service). Initially, poly (A)-containing mRNA molecules were purified from total RNA using poly (T) oligo-attached magnetic beads. Following the purification step, mRNA was fragmented into small pieces using divalent cations at elevated temperatures. The cleaved RNA fragments were then copied into first-strand cDNA fragment using reverse transcriptase and a high concentration of random hexamer primers. This was followed by a second-strand cDNA synthesis using DNA polymerase I and RNaseH. Finally, the short cDNA fragments were prepared for Solexa sequencing on Illumina HiSeq™ 2000, using the manufacturer’s protocol and reagents of the genomic DNA sequencing sample prep kit. The read lengths were 49 bp for leaf samples of both cotton lines.

The Illumina/Solexa approach involved sequencing of cDNA fragments and counting the number of particular fragments. The terminators were labeled with fluorescent compounds of four different colors to distinguish among the different bases at the given sequence position. The template sequence of each cluster was deduced by reading the color at each successive nucleotide addition step. We obtained 12.5 and 12.2 million reads from Illumina/Solexa sequencing for K1 and K2 cotton lines, and deposited them in Sequence Read Archive (SRA) database of GenBank under the accession no. SRR867611. At present, the full genome sequence for cotton is not available. The overall approach to non-model organism transcriptome analysis (Fig. S1) was to use high-throughput short-read sequences, optimize assembly and mapping parameters using partial data, and then process the total data using these optimized mapping and de novo parameters. Assembled contigs were compared with themselves and with the nominated reference EST using BLAST, leading to the extraction of candidate duplicate genes [53]. The results were visualized at different stages for validation purposes [54].

### Gene Annotation

mRNA and EST of cotton (ftp://ftp.ncbi.nih.gov/repository/UniGene/Gossypium_ hirsutum/Ghi.seq.uniq.gz) were used as a reference sequence to align and identify the sequencing reads. To map the reads to the reference, the alignments and the candidate gene identification procedure were conducted using the mapping and assembly with qualities software package [55]. This was done essentially by following the protocols described in the online documentation (http://maq.sourceforge.net) and adopting the default parameter values. Briefly, the reference sequences were converted to binary FASTA format, and each Solexa read data subset (corresponding to one lane on the instrument) was transformed from Solexa FASTQ to Sanger FASTQ format. As recommended, each subset was separately mapped to the reference, and these maps were then merged to form general maps for assembling the consensus sequences (Fig. S1) [53].

### Identification of Differentially Expressed Genes and Functional Analysis

To eliminate the influence of different gene length and sequencing level on the calculation, the RPKM method was used for normalization and the result can be directly used for comparing differences in gene expression between samples. The expression of genes was calculated by RPKM (Reads Per kb per Million reads) method [56] according to the formula:
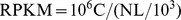
Where C is the number of reads that uniquely aligned to the gene, N is the total number of reads that uniquely aligned to all genes in the specific sample, and L is number of bases of the gene. The P-value corresponding to differential transcript expression in two samples was determined from Audic’s algorithm [57], and FDR (False Discovery Rate) method was applied to determine the threshold of P-values in multiple tests. We used ‘‘FDR ≤0.001 and the absolute value of log2Ratio ≥1” as the threshold to determine the significance of gene expression difference [58].

GO enrichment analysis was performed for functional categorization of differentially expressed transcripts using agriGO software [59] and the P-values corrected by applying the FDR correction to control falsely rejected hypotheses during GO analysis. The pathway analysis was conducted using KEGG (www.genome.jp/kegg/).

### Real-time PCR (RT-PCR) Analysis

The expression of some important genes was determined using RT-PCR. The leaves from 6 representative individual plants of each line were harvested and every 2 leaves were combined into one biological replicate and then extracted for total RNA using the TRIzol reagent (Invitrogen). A first-strand cDNA fragment was synthesized from total RNA using Superscript II reverse transcriptase (Invitrogen). Gene-specific primers were designed according to the gene sequences using the Primer Premier 5.0 (Premier Biosoft International, Palo Alto, CA) and then synthesized commercially (Shanghai Sangon Biological Engineering Technology & Services Co., Ltd., Shanghai, China). The primers are listed in Fig. S2. The amplification of β-actin was used as an internal control to normalize all data. 20 µL samples were run in triplicate on a Bio-red IQ2 Sequence Detection System and Applied Biosystems software using 0.1 µL first-strand cDNAs and SYBR Green PCR Master Mix (Applied Biosystems). Thermal cycling was performed at an initial denaturation step at 95°C for 3 min followed by 40 cycles at 95°C for 10 s, at annealing temperatures of 60°C for 10 s, and at 72°C for 10 s. Relative quantization of gene expression was calculated and normalized to β-actin.

## Results

Seedling emergence, squaring, flowering, peak flowering, peak boll-setting, and boll-opening for both K1 and K2 occurred at about 6, 40, 60, 75, 90, and 110 DAP. The growth and development of both lines followed a similar time-course and growth performance was considerably similar. However, leaf senescence based on chlorophyll content and photosynthesis in the late season differed significantly between the two lines (Fig. S3). The chlorophyll content and photosynthesis of K2 were considerably higher than those of K1 at 110 DAP.

### Solexa Sequencing and Assessment

We generated 12.5 and 12.2 million reads from one lane of Illumina/Solexa sequencing for K1 and K2 lines. Prior to mapping these sequencing reads to the reference mRNA and expressed sequence tag (EST), only adaptor reads, containing N reads and low-quality reads were filtered. After filtration, 12.4 and 12.1 million usable reads for K1 and K2 were obtained. Since the full genome sequence for cotton is not available, mRNA and EST contigs of cotton (ftp://ftp.ncbi.nih.gov/repository/UniGene/Gossypium_hirsutum/Ghi.seq.uniq.gz) were used as reference sequences to align and identify the sequencing reads. This allowed for the mapping of 44.01 and 44.19% of the K1 and K2 reads that passed our filters, representing about 5.45 and 5.36 million reads (Table 1).

**Table 1:** Total number of sequencing reads obtained from each sample.

	Early-senescence cotton line		Late-senescence cotton line	
Map to gene	Read number	Percentage	Read number	Percentage
Total good reads	12392329	100%	12135428	100.00%
Total mapped reads	5454055	44.01%	5362278	44.19%
unique match	5167284	41.70%	5091444	41.96%
multi-position match	286771	2.31%	270834	2.23%
Total Unmapped Reads	6938274	55.99%	6773150	55.81%

### Identification and Functional Classification of Differentially Expressed Genes

**Figure 1:**
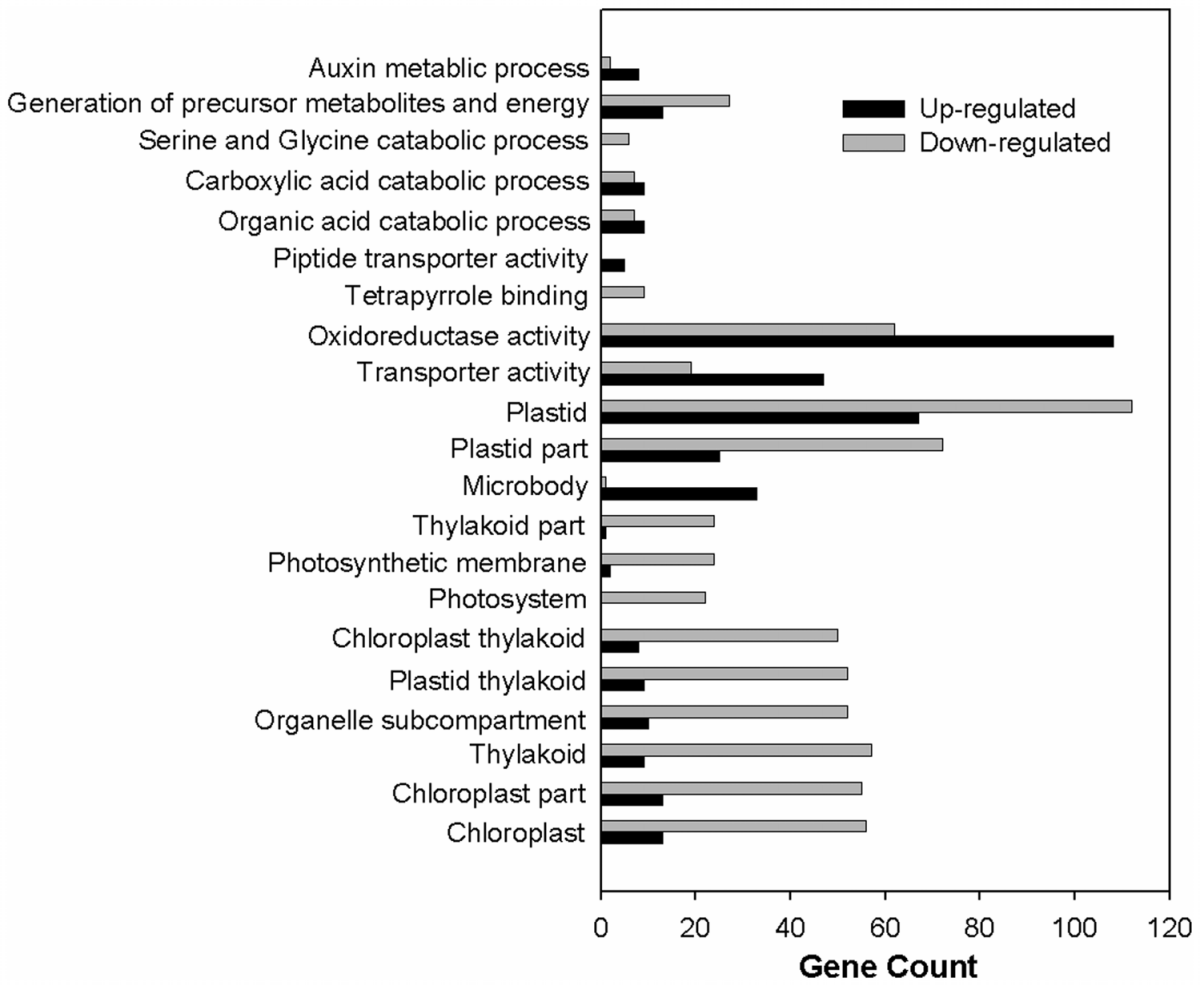
GO analysis of differentially expressed genes obtained from Solexa sequencing. The abscissa of the bar plot represents the gene count within each GO category. All processes listed had enrichment *p* values <0.05.

Putative differentially expressed genes were finally selected depending on the expression profiles and whether: a) the average fold change between K1 and K2 genes was more than or equal to twofold, and b) the FDR was less than 0.001. Accordingly, 1132 genes were identified as having been up-regulated in K1 compared with K2. Meanwhile, the expression of 455 genes was decreased by more than twofold in K1 compared with K2. Gene ontology (GO) analysis was performed by mapping each differentially expressed gene into the records of the GO database (http://www.geneontology.org/). The GO annotation of these genes is presented in Fig. 1. The main functional groups of up-regulated genes were related to auxin metabolic process, carboxylic acid and organic acid catabolic process, oxidoreductase activity, transporter activity and microbody; the main functional groups of down-regulated genes were related to serine and glycine catabolic process, generation of precursor metabolites and energy, tetrapyrrole binding, plastid and plastid part, thylakoid and thylakoid part, photosystem and photosynthetic membrane, organelle subcompartment and chloroplast and chloroplast part (Fig. 1).

To understand the functions of the differentially expressed genes, we mapped all the genes to terms in KEGG database and, compared this with the whole transcriptome background, with a view to searching for genes that were significantly enriched (Table 2). Among the differentially expressed genes with KEGG pathway annotation, 250 genes were attributed to the first significantly enriched metabolic pathway. There were 152 up- and 98 down-regulated genes in the metabolic pathway. Notably, the specific enrichment of metabolic pathway genes was observed to be involved in valine, leucine, isoleucine, lysine and tryptophan metabolism and degradation, alpha-linolenic acid metabolism, fatty acid metabolism, nitrogen metabolism, glutathione metabolism, glycerophospholipid metabolism, ascorbate and aldarate metabolism; more of the differently expressed genes were up-regulated in K1 than K2. Photosynthesis, photosynthesis-antenna proteins and carbon fixation in photosynthetic organisms pathways were also enriched in KEGG pathway analysis, with most of the differently expressed genes been down-regulated in K1 compared with K2. Plant-pathogen interaction pathway was another enriched pathway with most of the differentially expressed genes been significantly up-regulated in K1 compared with K2.

**Table 2:** KEGG pathway annotation of differentially expressed genes obtained from Solexa sequencing.

Pathways	DEGs with pathway annotation (783)	All genes with pathway annotation (9335)	Up-regulated gene (K1/K2)	Down-regulated gene (K1/K2)
**Metabolic pathways**	250	2227	152	98
**Alpha-linolenic acid metabolism**	22	72	14	8
**Fatty acid metabolism**	21	74	18	3
**Biosynthesis of secondary metabolites**	133	1141	87	46
**Photosynthesis**	17	78	0	17
**Biosynthesis of unsaturated fatty acids**	14	59	10	4
**Photosynthesis-antenna proteins**	7	19	0	7
**Peroxisome**	23	134	21	2
**Carbon fixation in photosynthetic organisms**	18	98	3	15
**Plant-pathogen interaction**	68	568	56	12
**Valine, leucine and isoleucine degradation**	11	51	10	1
**Glyoxylate and dicarboxylate metabolism**	9	39	6	3
**Limonene and pinene degradation**	14	81	11	3
**Flavonoid biosynthesis**	19	124	11	8
**Indole alkaloid biosynthesis**	5	16	3	2
**Glycosphingolipid biosynthesis globo series**	3	6	3	0
**Steroid biosynthesis**	8	38	2	6
**Phenylpropanoid biosynthesis**	25	189	15	10
**Nitrogen metabolism**	10	56	7	3
**Lysine degradation**	6	26	6	0
**Glutathione metabolism**	14	91	11	3
**Vitamin B6 metabolism**	5	20	3	2
**Glycolysis/Gluconeogenesis**	20	150	10	10
**Glycerophospholipid metabolism**	14	95	10	4
**Tryptophan metabolism**	7	36	7	0
**Stilbenoid, diarylheptanoid and gingerol biosynthesis**	14	96	9	5
**Ascorbate and aldarate metabolism**	12	84	9	3

All pathways listed had enrichment *p* values <0.05.

### Hormone Related Genes and Transcription Factor Genes

There were 51 differently expressed hormone-related genes, of which 39 were up-regulated and 12 down-regulated in K1 compared with K2 (Table 3). Isopentenyltransferase (*IPT*, Gene ID: DW494123) which is involved in cytokinin biosynthesis was down-regulated in K1 compared with K2. JA is critically involved in senescence. There are 6 differentially expressed JA-related genes, 5 of which were up-regulated in K1 compared with K2. Four of the 5 up-regulated genes were JA biosynthesis genes. Two differentially expressed SA related genes were both down-regulated in K1 compared with K2. In contrast to SA related genes, the 5 ethylene and 3 BR related genes were all up-regulated in K1 compared with K2. Eighteen of the 20 differently expressed auxin-related genes were up-regulated in K1 compared with K2. As for the 11 differently expressed GA-related genes, 8 genes were up-regulated and 3 down-regulated in K1 compared with K2. Furthermore, 2 GA biosynthesis genes, gibberellin 20-oxidase (gene ID: DQ122188) and Gibberellin 3-hydroxylase (gen ID: ES800765) were up-regulated but the GA degradation gene, GA 2-oxidase (gene ID: DW485236) was down-regulated in K1 compared with K2 (Table 3). Unexpectedly, only one ABA-related gene was up-regulated while 3 were down-regulated in K1 compared with K2. Moreover, the expression level of ABA biosynthesis gene *NCED* (9-cis-epoxycarotenoid dioxygenase, gene ID: EX168449) in K2 was 44 folds higher than that in K1.

**Table 3:** Differently expressed hormone related genes identified using Solexa sequencing in cotton.

Hormone	Gene ID	log_2_ Ratio(K1/K2)	*P*-value	Blast nr (annotation)
**Cytokinin**	DW494123	−1.61	<0.01	*Gossypium hirsutum* Isopentenyltransferase [*Gossypium hirsutum*]
**JA**	DT466567	3.09	<0.01	JA and ethylene-dependent systemic resistance, zinc finger protein [*Ricinus communis*]
	DW482657	1.66	<0.01	JA biosynthetic process, AMP dependent CoA ligase [Ricinus communis]
	ES813117	1.59	<0.01	JA biosynthetic process, 4-coumarate-coa ligase [*Populus trichocarpa*]
	ES850570	1.12	<0.01	JA biosynthetic process, AMP dependent CoA ligase [*Ricinus communis*]
	HM462002	1.06	<0.01	JA biosynthetic process, 0/3-hydroxyacyl-CoA dehyrogenase [*Ricinus communis*]
	DT548121	−1.16	<0.01	JA biosynthetic process, AMP dependent CoA ligase [*Ricinus communis*]
**SA**	EX165509	−1.63	<0.01	Response to salicylic acid stimulus, fiber protein Fb31 [*Gossypium barbadense*]
	ES828698	−1.54	<0.01	Salicylic acid-binding protein 2 [*Nicotiana tabacum*]
**Ethylene**	DT466567	3.09	<0.01	JA and ethylene-dependent systemic resistance, zinc finger protein [*Ricinus communis*]
	DW503700	1.64	<0.01	Ethylene-responsive transcription factor 2
	DW504358	1.45	<0.01	Response to ethylene stimulus, unknown [*Glycine max*]
	EX172462	1.18	<0.01	Response to ethylene stimulus, Methylthioribose kinase [*Ricinus communis*]
	ES804001	1.07	<0.01	Ethylene response factor 11 [*Actinidia deliciosa*]
**Auxin**	DT466567	3.09	<0.01	Auxin mediated signaling pathway, zinc finger protein [*Ricinus communis*]
	DW476189	2.15	<0.01	Auxin metabolic process, mitochondrial substrate carrier family protein [*Arabidopsis*]
	DW488782	1.95	<0.01	Response to auxin stimulus, LRR-repeat protein [*Ricinus communis*]
	EX166666	1.82	<0.01	Auxin metabolic process, peroxisomal membrane protein pmp34, [*Ricinus communis*]
	AI731637	1.73	<0.01	Auxin-regulated protein, SAUR family protein [*Populus trichocarpa*]
	DW482657	1.66	<0.01	Auxin metabolic process, AMP dependent CoA ligase [*Ricinus communis*]
	ES813117	1.59	<0.01	Auxin metabolic process, 4-coumarate-coa ligase [*Populus trichocarpa*]
	CO498303	1.59	<0.01	Auxin-responsive family protein [*Arabidopsis lyrata subsp. lyrata*]
	DT552463	1.55	<0.01	Auxin-induced in root cultures protein 12 precursor [*Ricinus communis*]
	ES816485	1.36	<0.01	Auxin metabolic process, dehydrogenase/reductase family protein [*Glycine max*]
	DW497941	1.30	<0.01	Auxin-responsive protein IAA1 [*Ricinus communis*]
	ES852227	1.20	<0.01	Auxin transport, aminopeptidase [*Arabidopsis thaliana*]
	DW500898	1.15	<0.01	Auxin metabolic process, anthranilate synthase, beta subunit, ASB1 [*Populus trichocarpa*]
	DT568077	1.15	<0.01	Response to auxin stimulus, carnitine racemase [*Ricinus communis*]
	DW499567	1.15	<0.01	Auxin : hydrogen symporter [*Ricinus communis*]
	ES808260	1.15	<0.01	Auxin-regulated protein [*Populus tremula* x *Populus tremuloides*]
	ES850570	1.12	<0.01	Auxin metabolic process, AMP dependent CoA ligase [*Ricinus communis*]
	EF467065	1.10	<0.01	*Gossypium hirsutum* auxin response factor 3 (ARF3)
	DT548121	−1.16	<0.01	Auxin metabolic process, AMP dependent CoA ligase [*Ricinus communis*]
	ES837803	−1.03	<0.01	Auxin metabolic process, nodulin-like protein [*Arabidopsis thaliana*]
**GA**	ES812534	2.47	<0.01	Gibberellin 3-beta-dioxygenase [*Ricinus communis*]
	ES800765	2.24	<0.01	Gibberellin 3-hydroxylase 1 [*Gossypium hirsutum*]
	DQ829776	1.98	<0.01	*Gossypium hirsutum* gibberellic acid receptor
	DT551192	1.47	<0.01	Gibberellin receptor GID1 [*Ricinus communis*]
	EF607794	1.32	<0.01	*Gossypium hirsutum* gibberellic acid receptor-b
	DQ122188	1.24	0.02	*Gossypium hirsutum* gibberellin 20-oxidase
	ES826231	1.06	<0.01	Gibberellin 3-beta-dioxygenase [*Ricinus communis*]
	FJ790128	1.01	0.02	Gibberellic acid mediated signaling pathway, GID1-4 [*Gossypium hirsutum*]
	DW485236	−3.48	<0.01	Gibberellin 2-oxidase [*Populus trichocarpa*]
	FJ384629	−1.72	<0.01	Gibberellin mediated signaling pathway, tonoplast intrinsic protein [*Gossypium hirsutum*]
	ES807419	−1.29	<0.01	Gibberellin mediated signaling pathway, unnamed protein product [*Vitis vinifera*]
**BR**	DT466203	4.44	<0.01	Brassinosteroid insensitive 1-associated receptor kinase 1 [*Ricinus communis*]
	ES794445	2.55	<0.01	Brassinosteroid-6-oxidase [*Vitis vinifera*]
	ES811041	1.52	<0.01	Brassinosteroid insensitive 1-associated receptor kinase 1 precursor, [*Ricinus communis*]
**ABA**	EX170109	1.07	<0.01	Abscisic acid responsive elements-binding protein 2 [*Populus trichocarpa*]
	EX168449	−5.48	<0.01	9-cis-epoxycarotenoid dioxygenase [*Ricinus communis*]
	ES836088	−2.21	0.02	Response to abscisic acid stimulus, unnamed protein product [*Vitis vinifera*]
	EX165509	−1.63	<0.01	Response to abscisic acid stimulus, fiber protein Fb31 [*Gossypium barbadense*]

It was also noted that 48 TFs including 11 NAC domain proteins were differentially expressed during leaf senescence (Table 4, 5). Eight WRKY and 3 R2R3-myb TFs along with 5 of the 6 differentially expressed ERF TFs were up-regulated in K1 compared with K2. Interestingly, the 11 differentially expressed NAC domain proteins were all up-regulated in K1 compared with K2, suggesting that NAC domain proteins may have important roles in cotton leaf senescence (Table 5).

**Table 4:** Differently expressed transcription factor genes identified using Solexa sequencing in cotton (K1/K2).

Transcription factor	Gene ID	log_2_ Ratio(K1/K2)	*P*-value	Blast nr (annotation)
**ERF**	DW233991	3.92	<0.01	ERF transcription factor 4 [*Vitis pseudoreticulata*]
	DT468163	2.78	0.02	AP2/ERF domain-containing transcription factor [*Populus trichocarpa*]
	DW227971	2.60	<0.01	AP2/ERF domain-containing transcription factor [*Populus trichocarpa*]
	DW503700	1.64	<0.01	Ethylene-responsive transcription factor 2
	DT554469	1.13	<0.01	AP2/ERF domain-containing transcription factor [*Populus trichocarpa*]
	DW236983	−1.90	<0.01	AP2/ERF domain-containing transcription factor [*Populus trichocarpa*]
**WRKY**	DT463609	2.39	<0.01	WRKY transcription factor 16 [*Populus tomentosa* x *P. bolleana*)]
	DT562541	2.04	<0.01	WRKY transcription factor 33 [*Populus tomentosa* x *P. bolleana*)]
	DT468618	1.90	<0.01	WRKY transcription factor [*Ricinus communis*]
	DT544987	1.85	<0.01	WRKY transcription factor [*Ricinus communis*]
	DW240842	1.37	<0.01	WRKY transcription factor [*Ricinus communis*]
	ES817646	1.36	<0.01	WRKY transcription factor [*Ricinus communis*]
	EX168442	1.35	<0.01	WRKY transcription factor 1 [*Populus tomentosa* x *P. bolleana*)]
	ES839303	1.23	<0.01	WRKY Transcription factor 1 [*Gossypium arboreum*]
**R2R3-myb**	ES808205	2.20	<0.01	R2R3-myb transcription factor [*Ricinus communis*]
	ES820794	1.24	<0.01	R2R3-myb transcription factor [*Ricinus communis*]
	ES821169	1.20	<0.01	R2R3-Myb transcription factor [*Citrus sinensis*]
**Putative TF**	ES842342	2.30	0.04	Transcription factor, putative [*Ricinus communis*]
	EX165732	1.43	<0.01	Transcription factor, putative [*Ricinus communis*]
	ES844147	1.11	<0.01	Transcription factor, putative [*Ricinus communis*]
	ES829573	1.04	<0.01	Transcription factor, putative [*Ricinus communis*]
	DW500579	−1.62	<0.01	Transcription factor, putative [*Ricinus communis*]
	DW503784	−1.09	<0.01	Transcription factor, putative [*Ricinus communis*]
	DT463358	−1.06	<0.01	Transcription factor, putative [*Ricinus communis*]
**Heat stress**	EX166632	1.62	<0.01	Heat stress transcription factor [*Carica papaya*]
	ES800773	1.08	<0.01	Heat stress transcription factor [*Carica papaya*]
**BZIP**	DR454207	1.60	<0.01	BZIP domain class transcription factor [*Malus* x *domestica*]
**GRAS**	DT051524	1.41	<0.01	GRAS family transcription factor [*Populus trichocarpa*]
**GATA**	ES803228	1.36	<0.01	GATA transcription factor [*Ricinus communis*]
**TCP**	ES817176	1.33	<0.01	TCP domain class transcription factor [*Malus* x *domestica*]
**TGA**	ES848366	1.25	<0.01	Transcription factor TGA7 [*Ricinus communis*]
**CCAAT-binding**	ES841782	1.10	<0.01	CVAAT-binding transcription factor [*Ricinus communis*]
	ES822081	1.09	0.06	CVAAT -binding transcription factor subunit A [*Ricinus communis*]
**MYB**	DW481287	−1.81	<0.01	MYB transcription factor MYB127 [*Glycine max*]
**MYBR**	DW238551	−1.14	<0.01	MYBR domain class transcription factor [*Malus* x *domestica*]
**BIM**	DW508557	−1.49	<0.01	Transcription factor BIM1 [Ricinus communis]
**ARF**	ES834779	−1.33	<0.01	ARF domain class transcription factor [*Malus* x *domestica*]

**Table 5:** Differently expressed NAC genes identified using Solexa sequencing in cotton.

Gene ID	log_2_ Ratio (K1/K2)	*P*-value	Blast nr (annotation)
DW517699	3.07	<0.01	NAC domain protein NAC6 [*Gossypium hirsutum*]
DR458413	2.75	<0.01	NAC domain-containing protein 21/22 [*Ricinus communis*]
CA992724	2.21	<0.01	NAC domain-containing protein [*Ricinus communis*]
EU706340	2.20	<0.01	*Gossypium hirsutum* NAC domain protein NAC6
EU706342	2.06	<0.01	*Gossypium hirsutum* NAC domain protein NAC4
CA992692	1.84	<0.01	NAC domain-containing protein [*Ricinus communis*]
ES806311	1.62	<0.01	NAC domain-containing protein [*Ricinus communis*]
EU372996	1.45	<0.01	NAC domain-containing protein [*Ricinus communis*]
EX166759	1.21	<0.01	NAC domain protein [*Citrus trifoliata*]
EU706343	1.68	<0.01	*Gossypium hirsutum* NAC domain protein NAC5
ES839947	1.09	<0.01	NAC transcription factor [*Vitis pseudoreticulata*]

### Confirmation of Solexa Expression Patterns by RT-PCR Analysis

**Figure 2:**
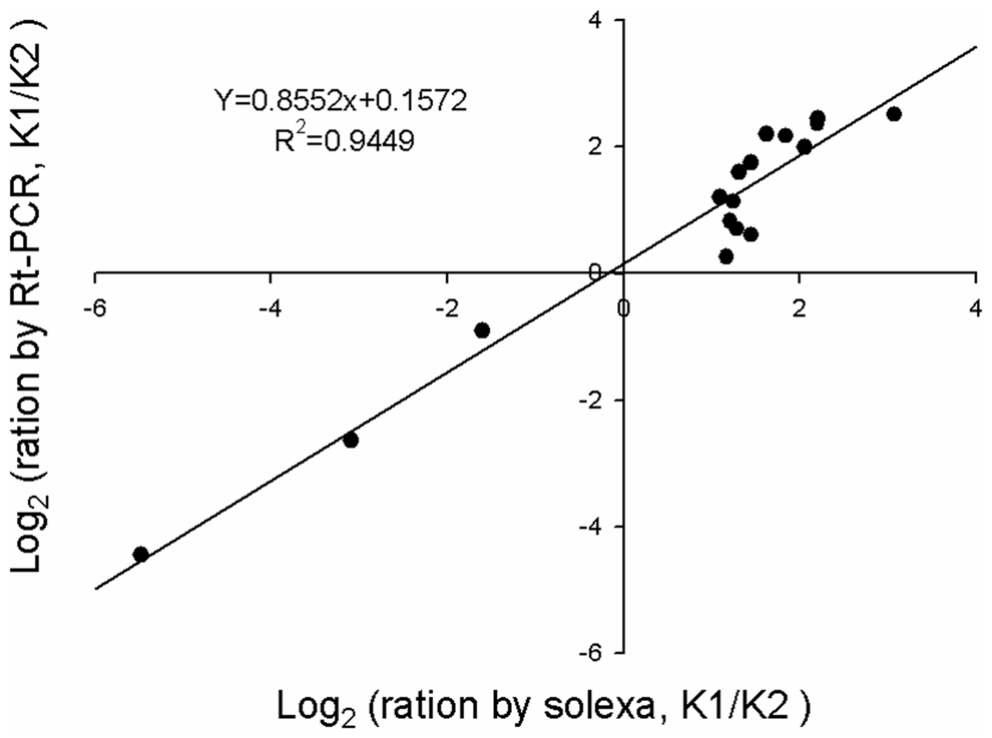
Comparison of the expression ratios of some selected genes using Solexa sequencing and qRT-PCR.

To test the reliability of Solexa sequencing further, RT-PCR analysis was performed with specific primers for a subset of 18 genes, which have been identified by Solexa sequencing in which 14 genes were up-regulated and 4 genes down-regulated. The results showed that 17 of the 18 genes had the same expression profiles as the original Solexa sequencing. This indicates that the original Solexa pattern was validated in 94.4% of the cases. This was not the case for the other gene presumably because of the mutations within the primer sites or because the RNA used for Solexa sequencing and qRT-PCR were extracted from different plants. The expression patterns of the 17 genes were highly consistent with the Solexa sequencing ratios, with a relative *R^2^* of 0.9449 (Fig. 2).

**Figure 3:**
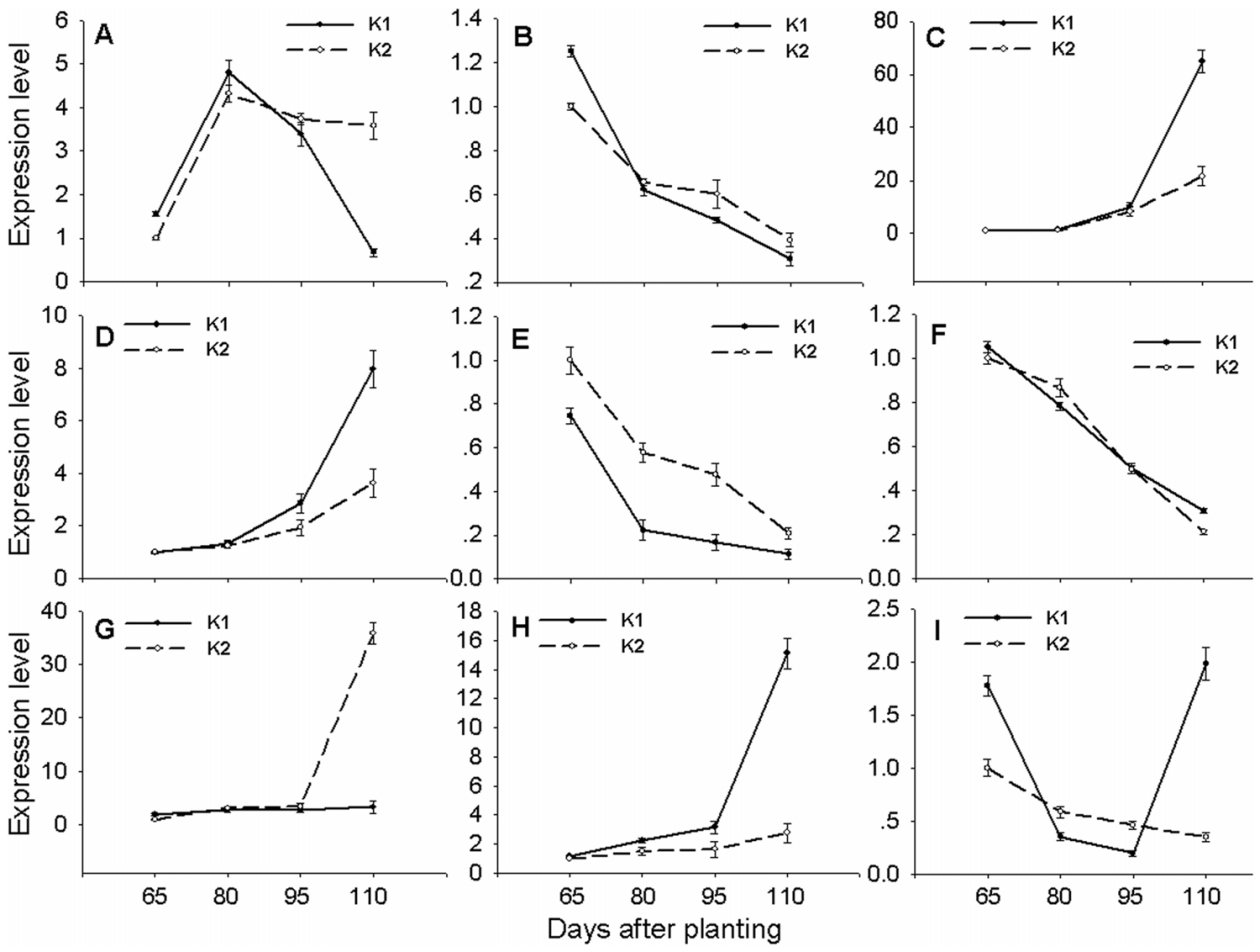
Senescence-dependent changes in gene expression determined by quantitative RT-PCR in the main-stem leaves of K1 and K2 cotton lines at 65, 80, 95 and 110 DAP. A, Chlorophyll binding protein (*LHCB*; gene ID: EX169337). B, Large subunit of Rubisco, ribulose- 1,5-bisphosphate carboxylase/oxygenase (*RBCL*; gene ID: ES820978). C, Superoxide dismutase (*SOD*; gene ID: ES824305). D, Autophagy (*ATG*; gene ID: CO493577). E, Isopentenyltransferase (*IPT*; gene ID: DW494123). F, 9-cis-epoxycarotenoid dioxygenase (*GhNCED2*; gene ID: HM014161); G, 9-cis-epoxycarotenoid dioxygenase (*NCED1*; gene ID: EX168449). H, NAC domain protein (*NAC*; gene ID: CA992724). I, NAC domain protein (*GhNAC6*; gene ID: Dw517699). Expression ratios are presented relative to K2 values at 65 DAP. Data are means of three biological replicates ± SE.

To test if the differentially expressed genes were developmental stage-dependent, the expression patterns of 9 senescence-related genes were analyzed by RT-PCR at 65, 80, 95 and 110 DAP. For both K1 and K2, the expression level of *LHCB* (Chlorophyll binding protein, gene ID: EX169337) was increased gradually before 80 DAP but decreased after 80 DAP during leaf senescence and the *LHCB* expression level of K1 was distinctly lower than K2 at 110 DAP (Fig. 3A). The expression level of *RBCL* (large subunit of Rubisco, ribulose-1, 5-bisphosphate carboxylase/oxygenase; gene ID: ES820978) decreased as K1 and K2 plants started to senesce from 65 to 110 DAP and the *RBCL* expression level of K1 was lower than K2 at 80 and 110 DAP (Fig. 3B). The expression level of *SOD* (superoxide dismutase, Gene ID: ES824305) and *ATG* (autophagy, gene ID: CO493577) in K1 and K2 plants increased during senescence and *SOD* and *ATG* expression levels of K1 were distinctly higher than K2 at 95 and 110 DAP (Fig. 3C, D ). The *IPT* (isopentenyltransferase, gene ID: DW494123) expression level decreased as K1 and K2 started senescence and the level of K1 was lower than that of K2 from 65 to 110 DAP (Fig. 3E). The *GhNCED2* (gene ID: HM014161) expression level also decreased as K1 and K2 started to senesce but the level of K1 was higher than that of K2 at 110 DAP (Fig. 3F). However, the *NCED1* (gene ID: EX168449) expression level increased as K1 and K2 started to senesce but the level of K1 was lower than that of K2 at 110 DAP (Fig. 3G). The expression level of *NAC* (NAC domain protein, gene ID: CA992724) in K1 and K2 increased during senescence but that in K1 was higher than that in K2 from 65 to 110 DAP (Fig. 3H). On the contrary, the expression level of another NAC domain protein gene *GhNAC6* (gene ID: Dw517699) in K2 gradually decreased during senescence and that in K1 also gradually decreased before 95 DAP. The *GhNAC6* expression level was distinctly increased after 95 DAP, therefore, the level in K1 was higher than that in K2 at 110 DAP (Fig. 3I).

## Discussion

Early leaf senescence has become one of the important factors limiting cotton production in recent years [8], [60]. Understanding its causes and mechanisms would help reduce yield loss due to premature senescence. Leaf senescence varies greatly among cotton genotypes. Our previous study suggested that K1 aged earlier than K2 because of more cytokinins but less ABA in leaves of K1 than K2 [19]. In this study, the genome-wide changes in gene expression between K1 and K2 at 110 DAP were investigated by Illumina/Solexa sequencing method. We found that K1 differed significantly from K2 in the expression of senescence-related genes during leaf senescence. About 1132 genes were up-regulated and 455 genes down-regulated in K1 compared with K2.

Leaf senescence begins with the transition from anabolism of carbohydrates and other biomolecules to catabolism of proteins, nucleic acids, and lipids, and culminates in cell death. The onset and progression of leaf senescence is accompanied by expression of a large number of genes [11], [12]. Down-regulated genes are significantly enriched for genes linked to the plastid and thylakoid, and with functions in metabolic processes, particularly photosynthesis and carbohydrate and amino acid metabolism [10]. Genes involved in chloroplast activity such as photosystem (PS) I and II, carbon fixation, chlorophyll (tetrapyrrole) biosynthesis, and amino acid metabolism are also down-regulated during senescence [10]. In our experiment, 1132 genes were up-regulated and 455 genes down-regulated in K1 compared with K2. Down-regulated genes were related to serine and glycine catabolism, tetrapyrrole binding, plastid and thylakoid, photosystem and photosynthetic membrane, organelle subcompartment, chloroplast and chloroplast part, carbon fixation and steroid biosynthesis (Fig. 1 and table 2). The senescence-repressed gene, *LHCB1* has been used to monitor the progress of senescence in *otsB*-expressing plants and the *LHCB1* expression level in *otsB*-expressing plants was higher than the wild type plants [61]. In the present study, the *LHCB* expression level in K1 was lower than in K2 at 95 and 110 DAP, which might be an attribute to earlier senescence in K1 than K2 (Fig. 3A). The levels of *RBCL* mRNA exhibited a gradual decrease which started before the onset of visible senescence in two different senescent types of maize and a decline in *RBCL* could lead to degradation of Rubisco, thereby decreasing the photosynthetic rate during leaf senescence [62], [63]. Therefore, in the present study the decreased chlorophyll content and photosynthesis in K1 might be attributed to reduced expression of *LHCB* and *RBCL* at 110 DAP (Fig. 3A, B and Fig. S3).

During senescence different pathways contribute to degradation of proteins and other macromolecules, of which autophagy (ATG) is an important pathway. The ATG plays a key role in the senescence process, and accelerated senescence has been observed in a number of autophagy-defective mutants [64]–[66]. Knockout or RNAi mutants of *AtATG4a/b*, *AtATG5*, *AtATG7*, *AtATG9*, and *AtATG18a* exhibit accelerated senescence and hypersensitivity to nutrient starvation, indicating that autophagy is not only essential for the senescence program but also plays an important role in nutrient remobilization [64]–[68]. A key role of senescence in plant tissues is the ordered degradation of macromolecules and mobilization of the products, during which supposedly transporters (TPs) are critically involved. There are many Arabidopsis TPs which are up-regulated during senescence [11], [12], [46], [69], [70]. The preponderance of up-regulated TPs corresponds well with the substrates (amino acids, inorganic phosphorus, sugars, purines, pyrimidines and metal ions), which are known to be transported from senescent leaves to sink organs [71], [72]. The up-regulation of amino acid and oligopeptide TPs correlates with the increase in protein degradation, and the breakdown products were exported to the sink organs by TPs during senescence [9]. The expression levels of protein degradation, Peroxisome, ATG and TP related genes in K1 were higher than those in K2, indicating that proteins and other macromolecules degrade and then export the nutrients to sink organs much earlier in K1 than in K2 (Fig. 1, Fig. 3D and Table 2).

Cytokinin is involved in leaf senescence, because its levels are reduced in senescing leaves [6], [19] and exogenous application of cytokinin or endogenous overexpression of *IPT* gene can delay senescence [22], [73]. In this study, the expression level of *IPT* in both K1 and K2 were gradually decreased, but he *IPT* level in K1 was distinctly lower than K2 from 65 to 110 DAP (Fig. 3E and table 3). The results well matched our previous observation that the cytokinin contents in both K1 and K2 were gradually decreased, but the cytokinin contents in K1 were distinctly lower than in K2 from 65 to 110 DAP. This implied that the decreased cytokinin contents in K1 and K2 may be caused by decreased *IPT* expression level [19]. Thus it was suggested that enhanced leaf senescence and reduced cytokinin level of K1 was attributed to decreased *IPT* expression in K1 relative to K2. On the contrary, ABA is considered a promoter of senescence because ABA treatment can induce leaf senescence and many genes related to ABA synthesis, metabolism and signaling are up-regulated during senescence [10]–[12], [14], [74], [75]. However, not all ABA-response genes were up-regulated during senescence. Therefore, the estimate of the impact of ABA as a regulator during senescence is not straightforward [12]. There are many *NCED* gene members and different *NCED* genes have different temporal- and tissue-specific expression patterns [76]–[78]. The expression of the *CsNCED1* gene was consistent with the accumulation of ABA but the *CsNCED2* gene showed a different and tissue-specific expression during fruit maturation [77]. In our study, 3 of the 4 differently expressed ABA-related genes were down-regulated in K1 compared with K2, though K1 senescence earlier than K2 (Table 3). One *NCED* (EX168449) was gradually increased but another *GhNCED* (HM014161) was gradually decreased from 65 to 110 DAP during senescence and the expression level of *NCED* (EX168449) in K1 was considerably lower than in K2. However, the *GhNCED* (HM014161) expression level in K1 was distinctly higher than K2 at 110 DAP (Fig. 3F, G). The expression of *NCED* genes was not consistent with our previous observation that the ABA level in K1 and K2 were gradually increased during senescence and K1 was higher than K2 in ABA level [19]. Such inconsistency might be due to the fact that the 2 *NCED* genes were not exactly the senescence-related genes or ABA was not a straightforward senescence regulator in K1 and K2 cotton plants. Further study on the exact roles of *NCED* genes may help better explain such inconsistency.

Although auxin effects on leaf abscission were first reported more than 50 years ago, its involvement in leaf senescence is much less understood than that of ET, JA, or cytokinin. In senescing leaves of Arabidopsis, the indole-3-acetic acid (IAA) concentration was 2-fold higher than in non-senescing leaves [37]. However, auxin treatment leads to a transient decrease in *SAG12* expression [30]. Arabidopsis plants over-expressing the auxin biosynthesis gene *YUCCA6*, such as the yuc6-1D activation mutant and *35S:YUC6* transgenic plants, have been shown to contain an elevated level of free IAA and reduced transcript abundances of *SAG12*, *NAC1*, and *ORE1* during leaf senescence compared with the wild type. It was suggested that auxin delayed senescence by directly or indirectly regulating the expression of senescence-associated genes [34]. In contrast to K2, 18 of the 20 differentially expressed auxin related genes were up-regulated in K1, suggesting that auxin might play an important role in leaf senescence of cotton (Table 3). The exact roles of auxin and the differentially expressed gene effects on cotton senescence need further study.

It is still not well determined if GA activity is involved in leaf senescence. GA can partially inhibit the cytokinin effect from seedling development to senescence through SPINDLY protein [39]. No GA biosynthesis gene but GA-inducible GA 2-oxidase 2 (*AtGA2OX2*) that deactivates GA was up-regulated during leaf senescence, suggesting that at least some GAs are deactivated during leaf senescence [12]. On the contrary, the GA biosynthesis genes, gibberellin 20-oxidase (gene ID: DQ122188) and Gibberellin 3-hydroxylase (gen ID: ES800765) were up-regulated and GA 2-oxidase (gene ID: DW485236) down-regulated in K1 compared with K2, suggesting that GA content in K1 was higher than K2 (Table 3). In contrast to K2, the early senescence cotton line K1 had higher GA but lower cytokinin content, suggesting a regulatory pathway similar to Arabidopsis that GA inhibits cytokinin effect through SPINDLY-like protein. The plant hormones JA, ethylene and BR are well known as regulators of leaf senescence. Exogenous application of JA and ethylene can enhance senescence [13], [14], [20]. Many JA, ethylene and BR related genes were up-regulated during senescence in Arabidopsis [11], [12]. In this study, 4 JA biosynthesis genes were up-regulated in K1 compared with K2, indicating that JA plays an important role in enhanced leaf senescence of K1 (Table 3). All the differently expressed ethylene and BR related genes were up-regulated in K1 compared with K2, indicating that JA, ethylene and BR are possibly involved in enhancing leaf senescence in K1 (Table 3).

Many TFs are differentially regulated and the NAC and WRKY families are particularly rich in senescence-regulated TFs in many plant species during leaf senescence [10], [11], [46], [70], [79]–[81], suggesting that they are involved in leaf senescence. Forty-eight differentially regulated TFs were found in our experiment (Table 4, 5). Among the TFs, NAC and WRKY that rank the first and second in TF families might play important roles in leaf senescence of cotton, because both were up-regulated during leaf senescence. Although more than 20 of the 106 known NAC genes in Arabidopsis exhibit senescence-dependent expression, the distinct regulatory function with respect to senescence has only been reported for some members so far [43], [48]–[51]. NAC (*At NAP*, *At ORE1* and *AtORS1*) have been identified as nonredundant positive regulators of senescence, because inhibiting them individually delays leaf senescence [48]–[50]. A dual-function NAC gene *VNI2* was recently reported to integrate ABA signaling with leaf senescence and to negatively regulate xylem vessel formation [43]. A hydrogen peroxide induced NAC transcription factor *JUB1* gene was also reported to regulate Arabidopsis senescence [51]. Leaf aging was delayed in transgenic plants overexpressing the *VNI2 or JUB1* gene but significantly accelerated in a *VNI2* or *JUB1* deficient mutant, suggesting that *VNI2 and JUB1* are negative regulators of senescence [43], [51]. There were 11 up-regulated NAC TFs in our study. The expression level of *NAC* (gene ID: CA992724) in K1 and K2 was increased during senescence and that in K1 was higher than in K2 from 65 to 110 DAP (Fig. 3H). However, the expression level of *GhNAC6* (gene ID: Dw517699) in K1 and K2 gradually decreased before 95 DAP, although the level in K1 was distinctly increased after 95 DAP and that in K1 was higher than K2 at 110 DAP (Fig. 3I). The different expression patterns of both *NAC* genes in K1 and K2 during senescence suggest that different NAC TFs have different functions in leaf senescence of cotton. Further research on their exact functions in leaf senescence related candidate *NAC* genes is necessary.

Taken together, our results showed that K1 senescence earlier than K2. This is because most of the genes related to anabolism of carbohydrates and other biomolecules were down-regulated but those for catabolism of proteins, nucleic acids, lipids and nutrient recycle etc. were up-regulated in K1 compared with K2. The transition from anabolism to catabolism occurred earlier in K1 than K2, which might be regulated by up- or down-regulated hormone related and TF genes such as down-regulated cytokinin biosynthesis and GA degradation genes, up-regulated JA and GA biosynthesis genes and up-regulated NAC and WRKY genes in K1. Further studies on hormone related genes and NAC TFs will improve our understanding of the regulatory function of hormone and NAC TFs on leaf senescence.

## Supporting Information

Figure S1 **Overview of the short-read based transcriptome analysis approach for non-model organisms.** Mapping is an option only if a suitable reference genome is available; otherwise FASTA (or FASTQ) sequences must be used [54].
(TIF)

Figure S2 **Primers used for RT-PCR analyses.**
(TIF)

Figure S3 **Chlorophyll (Chl) content and net photosynthetic (Pn) rate of the fourth leaf from the apex on the main-stem at 65–110 d after planting.** Values are means ±SD (n = 4). Initial flowering, peak flowering, peak boll-setting, and initial boll-opening occurred at 65, 80, 95, and 110 d after planting.
(TIF)
