# Supplementary figures and images for: Gene Expression Profiles Deciphering Leaf Senescence Variation between Early- and Late-Senescence Cotton Lines
Source: PLoS One. 2013 Jul 29;8(7):e69847. doi: 10.1371/journal.pone.0069847 (PMC3726770; doi:10.1371/journal.pone.0069847)

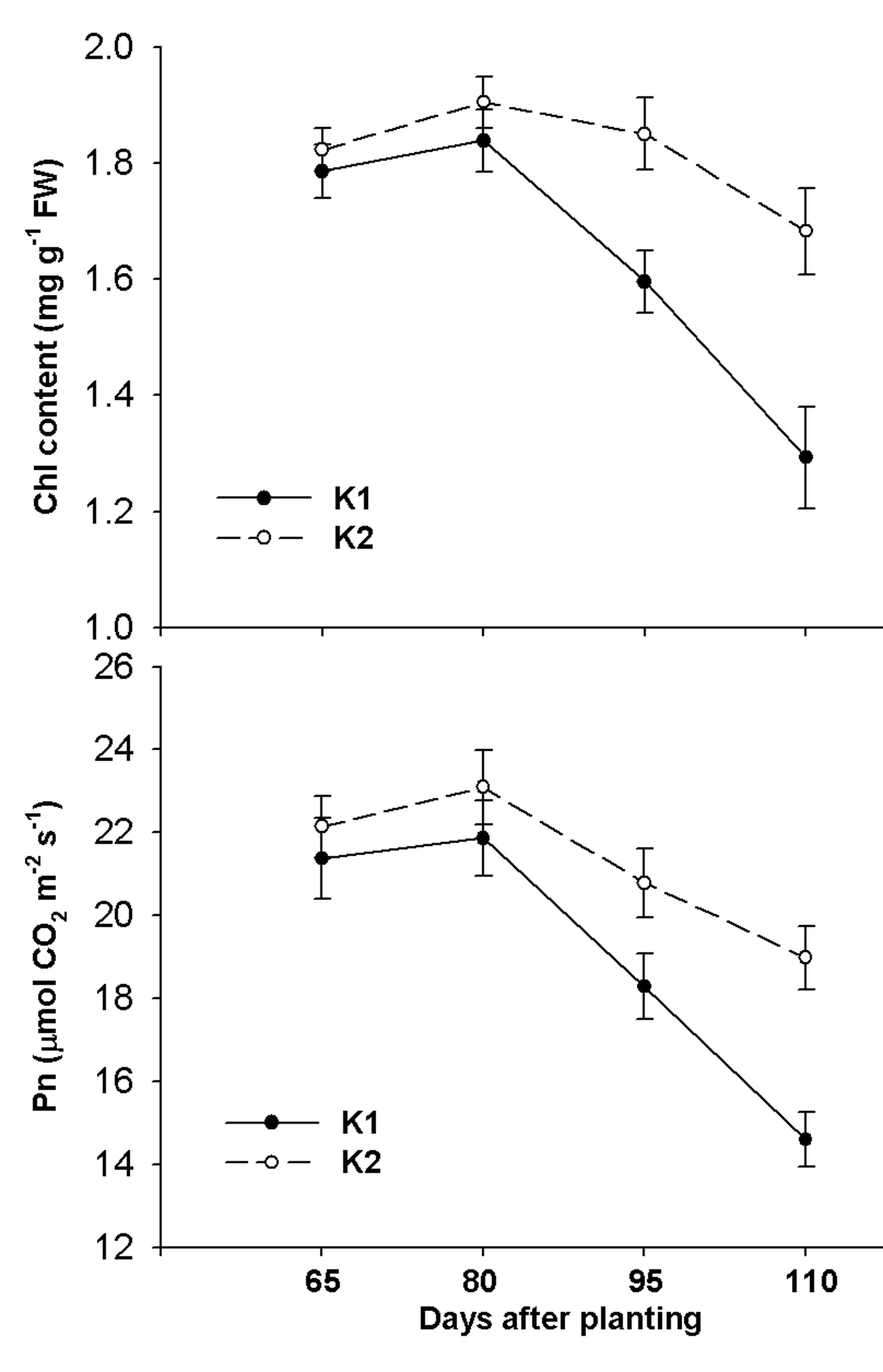

Supplement: Figure S3 — Chlorophyll (Chl) content and net photosynthetic (Pn) rate of the fourth leaf from the apex on the main-stem at 65–110 d after planting. Values are means ±SD (n = 4). Initial flowering, peak flowering, peak boll-setting, and initial boll-opening occurred at 65, 80, 95, and 110 d after planting. (TIF) [file pone.0069847.s003.tif]
